# Assessing Habitat Suitability and Range Dynamics of *Syzygium alternifolium* (Wight) Walp Under Future Climatic Scenarios

**DOI:** 10.1002/ece3.72861

**Published:** 2026-01-09

**Authors:** Abdul Rahim PP, Zishan Ahmad Wani, Javid Ahmad Dar, Subashree Kothandaraman, Yashwant S. Rawat

**Affiliations:** ^1^ Terrestrial Ecology and Modelling (TEaM) Lab, Department of Environmental Science and Engineering SRM University‐AP Amravati Andhra Pradesh India; ^2^ Centre for Geospatial Technology SRM University‐AP Amravati Andhra Pradesh India; ^3^ Department of Wood Technology Management, Faculty of Civil Technology Technical and Vocational Training Institute (TVTI) Addis Ababa Ethiopia

**Keywords:** conservation and management, MaxEnt, protected area, range change

## Abstract

*Syzygium alternifolium* (Wight) Walp is an ecologically and economically important tree species of dry deciduous forests of the Eastern Ghats. The species is classified as Endangered on the IUCN Red List under the criteria A2cd ver 3.1 due to declining populations threatened by habitat degradation and climate change. This research utilizes MaxEnt‐based species distribution modeling to assess its current and future habitat suitability under two future climate scenarios (SSP245 and SSP585) for the years 2050 and 2070, employing the MIROC6 global circulation model. The model demonstrated high performance (AUC = 0.93), with slope, precipitation of the warmest quarter (Bio18), and temperature of the wettest quarter (Bio8) identified as critical predictors. Currently, the extent of suitable habitats is limited (1262.39 km^2^), with 53.02% situated within protected areas. Projections suggest a steady increase in suitable area, potentially reaching up to 122.87% by 2070 (SSP585), although this expansion is significantly directed towards unprotected landscapes, indicating possible conservation gaps. These results underscore the necessity for proactive initiatives, including long‐term monitoring, eco‐physiological and genetic evaluations, and the incorporation of distribution modeling results into biodiversity action plans, forest management strategies, and regional climate adaptation frameworks to ensure the species' survival in light of future climate scenarios.

## Graphical abstract

We modeled the current and future habitat suitability of the endangered tree species *Syzygium alternifolium* using MaxEnt under two climate scenarios (SSP245 and SSP585) for 2050 and 2070. The model showed high accuracy (AUC = 0.93), with significant future range expansion projected outside protected areas, highlighting urgent conservation gaps. Our findings support integrating species distribution modeling into long‐term biodiversity and forest management strategies under climate change.

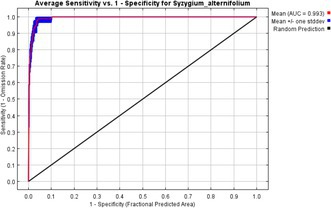

## 1 Introduction

Climate change is a significant and growing threat to global biodiversity, resulting in serious implications for ecosystem structure, species distributions, and the integrity of ecological functions and services (Onoh et al. 2024; Joshi et al. 2025). Rising temperatures, changes in rainfall patterns, an increase in the frequency of extreme weather events, and alterations in seasonality are disrupting ecological processes, which lead to habitat degradation, shifts in species ranges, declines in populations, and even the extinction of species (Ummenhofer and Meehl 2017; Muluneh 2021; Gupta et al. 2023). These effects are particularly severe for species with narrow ecological tolerances, limited dispersal capabilities, and restricted distributions (Wani et al. 2024). In this regard, identifying and accurately assessing suitable habitats under both current and future climatic conditions is essential for effective species management and conservation. Spatially explicit predictive tools, such as Species Distribution Models (SDMs), have gained importance in identifying suitable habitats, evaluating species vulnerability, and informing management strategies (Liu et al. 2022). SDMs connect species occurrence data with environmental variables to forecast habitat suitability across various spatial and temporal scales (Amindin et al. 2024). By projecting species distributions into future climate scenarios, SDMs provide valuable insights into potential species responses to environmental changes and assist in identifying future climate refugia, evaluating the effectiveness of protected areas, and supporting ex‐situ conservation efforts. These functionalities are especially crucial for rare and endemic species, which frequently lack adequate ecological research and face pressing conservation challenges. Among these tools, the Maximum Entropy model (MaxEnt) stands out as a robust method for modeling rare species, as it performs effectively even with limited sample sizes, accommodates categorical environmental variables, and requires only presence data (Mathur et al. 2023; Wang et al. 2024). It has been widely used to forecast species distributions under various climate change scenarios, assess conservation gaps, and plan habitat restoration (Yang et al. 2021; Khan et al. 2022; Roy et al. 2022; Lian et al. 2024; Xue et al. 2024).

**FIGURE 1:**
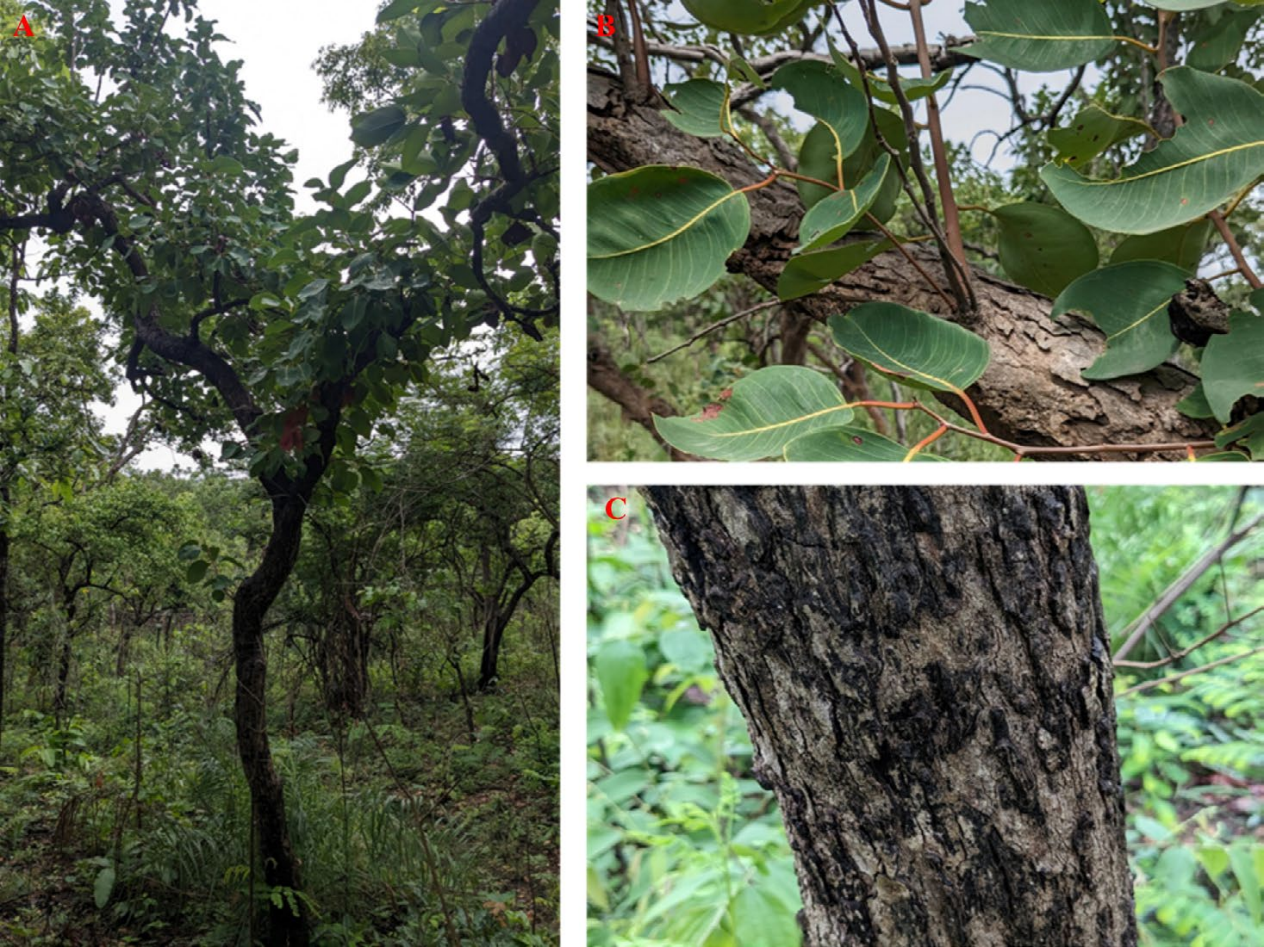
Morphological features of *Syzygium alternifolium* in its natural habitat. (A) a mature tree showing its erect, branched form; (B) close‐up of alternate, thick, and leathery leaves; (C) bark texture of a mature tree.

The genus *Syzygium* (Myrtaceae) consists of approximately 1200–1800 species that are found in tropical regions of Asia, Africa, and Australia (Uddin et al. 2022). In India, about 102 species of the genus *Syzygium* have been documented, with 44 of them being classified as endemic (Shareef and Kumar 2020). Among these endemic species is *Syzygium alternifolium* (Wight) Walp., which is located in the dry deciduous forests of the Eastern Ghats, particularly in the Seshachalam, Veligonda, and Lankamalla hill ranges in Andhra Pradesh, with isolated populations also noted in Tamil Nadu and Karnataka (Mohan and Lakshmi 2000; Pattanaik et al. 2022). *S. alternifolium* is a medium to large deciduous tree with smooth grayish bark, alternate thick leathery leaves, and white flowers borne in terminal cymes (Figure 1). It produces fleshy, dark purple berries containing a single large, hard seed. This species thrives in dry, rocky, slaty environments characterized by steep slopes, frequently forming gregarious stands in areas with minimal litter and low moisture levels (Raju et al. 2014). Locally, it is referred to as Mogi, Movi, Manchi, and Moyadi, and it possesses significant timber, medicinal, and ethnobotanical importance. Its seeds are utilized for diabetes treatment and have demonstrated hypoglycemic, antihyperglycemic, and antibacterial effects (Rao and Rao 2001; Kumar and Yasmeen 2013). The wood of this species is employed in construction. Despite its presence in protected areas such as the Sri Venkateshwara Wildlife Sanctuary, the species is currently under severe threat due to overharvesting, habitat degradation, and climate variability (Saha et al. 2015). Additionally, biological challenges such as erratic flowering, poor fruit set, predation of buds and fruits, and limited seed viability further exacerbate these pressures (Mohan and Lakshmi 2000). Given its limited distribution, ecological sensitivity, and high utility value, *S. alternifolium* is listed as Endangered under IUCN criteria A2cd ver 3.1 (Saha et al. 2015). Despite its critical status, there has been limited research into its ecological niche, environmental requirements, and future survival prospects under climate change. This study aims to fill this gap by utilizing the MaxEnt modeling framework to evaluate the habitat suitability of *S. alternifolium* in Andhra Pradesh, considering both current and anticipated climate scenarios. The study uses high‐resolution bioclimatic, topographic and soil data, coupled with species occurrence records, to model the species' ecological niche and forecast potential shifts in suitable habitats. The specific objectives of the study are to (a) Identify the key environmental variables influencing the current distribution of *S. alternifolium*, thereby understanding its ecological requirements and niche limitations; (b) Project the species' future habitat suitability under two Shared Socioeconomic Pathways (SSP245 and SSP585) for the time periods 2041–2060 and 2061–2080, and (c) Overlay predicted suitability maps with existing Protected Area boundaries, in order to evaluate how much of the suitable habitat currently and in the future lies within the protected area network and to identify areas outside protection that may require conservation attention. The study will provide a spatially explicit, climate‐informed strategy for the conservation of *S. alternifolium*. The findings are intended to support adaptive, evidence‐based interventions to ensure the long‐term survival of this endangered species, and offer a replicable framework for other rare taxa in the Eastern Ghats threatened by climate and anthropogenic change.

## 2 Materials and Methods

### 2.1 Study Area

**FIGURE 2:**
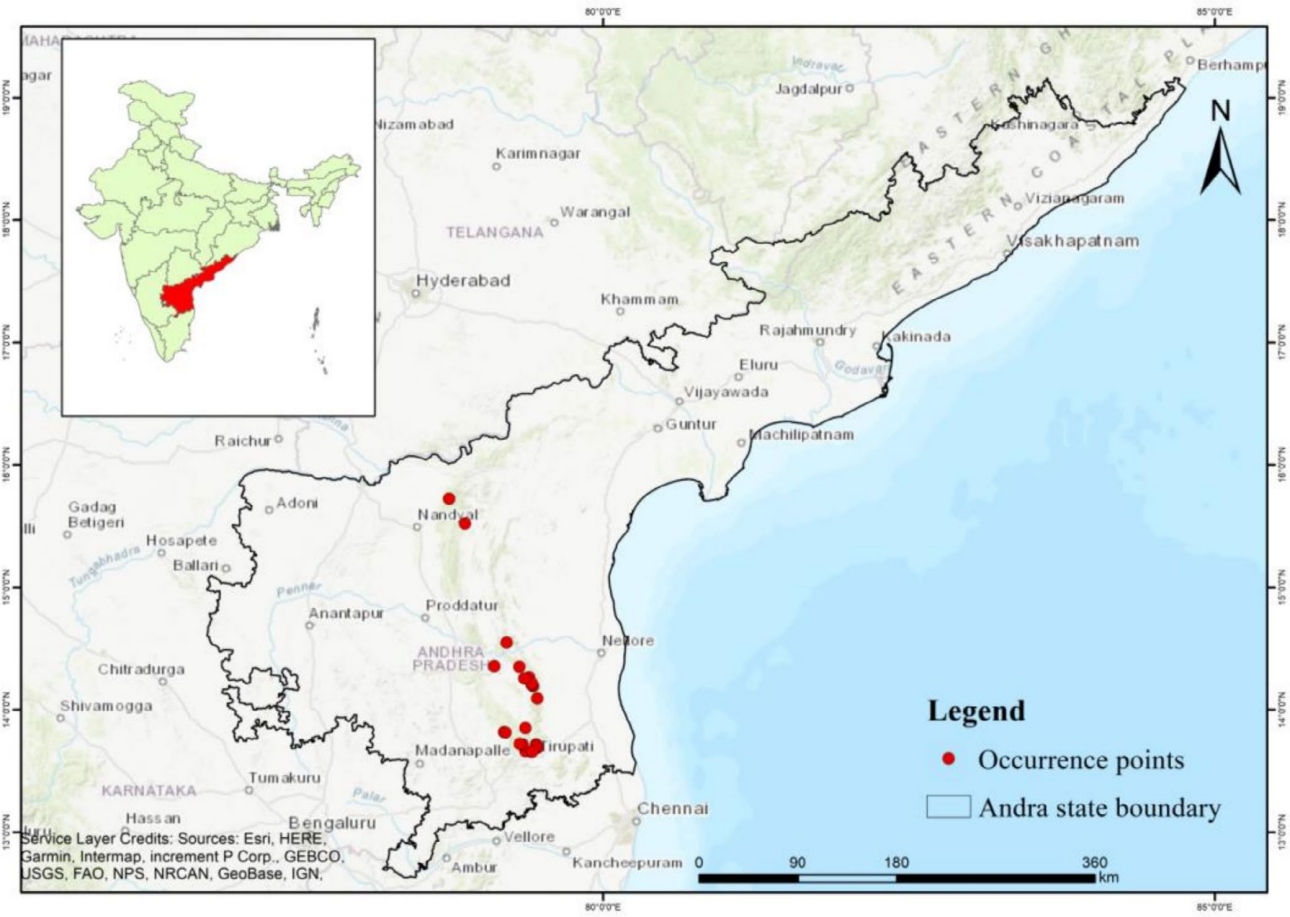
Map showing the location of study area along with the occurrence points of the targeted species.

Andhra Pradesh is an Indian state located in the eastern part of the Indian peninsula, lying between the latitudes 12°37′ N and 19°55′ N, and longitudes 76°45′ E and 84°46′ E (Figure 2). The state is bounded by Telangana to the north and northwest, Chhattisgarh and Odisha to the north and northeast, Tamil Nadu to the south, Karnataka to the west, and the Bay of Bengal to the east. The state covers an area of 275,068 km^2^. Its topography varies from sea level up to 1680 m (Pullaiah 2018). Geographically, it consists of coastal plains extending from the northern to the southern tip. The Eastern Ghats are a series of discontinuous hills that separate the coastal plains from the plateau, while the western peneplains feature scattered formations of hillocks. The state receives an average rainfall of 896 mm annually from both the southwest and northeast monsoons, with about 67% coming from the South‐West monsoon. During the summer, maximum temperatures range from 37°C to 44°C, while winter minimums range from 14°C to 19°C. Andhra Pradesh's three major rivers are the Krishna, Godavari, and Pennar (Reddy 2020). The region has six main soil types: red, black, alluvial, laterite, coastal sandy and skeletal soils. Forested areas cover 22,862 km^2^, constituting 14.27% of the total area. The forest types include tropical semi‐evergreen forests, tropical moist deciduous forests, southern dry deciduous forests, northern mixed dry deciduous forests, dry savannah forests, tropical dry evergreen forests, and tropical dry evergreen scrub (Pullaiah 2018). Biodiversity of the state is protected and managed with 14 protected areas.

### 2.2 Species Occurrence and Environmental Data

Occurrence data of *S. alternifolium* was compiled from two sources: primary field surveys conducted at known habitat areas and secondary from the Global Biodiversity Information Facility (GBIF) database (https://www.gbif.org/ assessed on 26 April, 2025). A total of 27 georeferenced occurrence points were collected, including 25 from primary field surveys and 2 from the GBIF. To minimize spatial sampling bias and overfitting in species distribution modeling (Luo et al. 2025), the records were spatially rarefied at a 1 km resolution using SDM toolbox in ArcGIS 10.8 following Pshegusov et al. (2022) and Munna et al. (2023). After spatial rarefying, 21 occurrence points (Table S1) were retained and used in the final model calibration.

A total of 19 bioclimatic variables representing temperature and precipitation variables were initially obtained from the WorldClim version 2.1 database (https://worldclim.org/ assessed on 29th April, 2025) at a spatial resolution of 30 arc‐seconds (~1 km) for the current baseline (1970–2000). In addition to climatic variables, topographic variables including elevation, slope, and aspect were also extracted from Shuttle Radar Topography Mission (SRTM) digital elevation model data downloaded from WorldClim. To incorporate edaphic factors, soil variables were downloaded from the Soil Grids database (https://soilgrids.org assessed on 30 April, 2025). Soil characteristics are fundamental determinants of plant growth, productivity, and reproductive success, as well as the relative performance of coexisting species and the structure and productivity of plant communities (Van der Putten et al. 2013). Inclusion of soil variables can significantly improve the predictions of plant species distribution (Buri et al. 2017). All raster layers were resampled to match the spatial resolution and extent of the study area. To reduce multicollinearity and retain ecologically meaningful predictors (Sillero and Barbosa 2021; Baker et al. 2022), a Pearson correlation analysis was performed using the ‘cor()’ function in R software. Variables with a correlation coefficient *r* > 0.7 were excluded (Table S2). Based on this screening, a final set of 12 environmental variables viz., Bio2 (Mean Diurnal Range), Bio8 (Mean Temperature of Wettest Quarter), Bio10 (Mean Temperature of Warmest Quarter), Bio11 (Mean Temperature of Coldest Quarter), Bio18 (Precipitation of Warmest Quarter), slope, elevation, aspect, soil total nitrogen mean (band 2), soil organic carbon density (band 2), and soil clay content (band 1 and 2) were selected for model development. Future climate projections were sourced from WorldClim v2.1 using the MIROC6 global circulation model (GCM) under two Shared Socioeconomic Pathways (SSPs): SSP245 (moderate emission scenario) and SSP585 (high emission scenario), for two future time periods: 2041–2060 (2050) and 2061–2080 (2070). The variables were downloaded at the same spatial resolution (30 arc‐seconds) and processed using the same set of predictors selected for the current scenario to ensure comparability across temporal scenarios.

### 2.3 Species Distribution Modeling

Species distribution modeling was conducted using the Maximum Entropy algorithm (MaxEnt version 3.4.4), a machine‐learning technique well‐suited for presence‐only data and small sample sizes (Rahimian Boogar et al. 2019; Fitzgibbon et al. 2022). We used auto features to run the algorithm, which optimizes model complexity and performance by enabling MaxEnt to automatically choose relevant feature classes based on the quantity of occurrence records. To evaluate the accuracy and stability of the model, we employed a bootstrap with 10 replicate runs, 10,000 background points, 1500 iterations, a default prevalence of 0.5, and 10th percentile training presence threshold. 20% of the occurrence records were used for testing, and 80% were used for training the model. The jackknife method was employed to assess the relative importance of each predictor variable. This approach evaluates model performance both when each variable is used in isolation and when it is excluded from the model, providing insight into each variable's unique contribution (Mahmoud et al. 2025; Wang et al. 2025). This allowed identification of the most influential environmental factors driving species distribution patterns. Model performance was evaluated using the Area Under the Curve (AUC) of the Receiver Operating Characteristic (ROC) curve. AUC is a robust, threshold‐independent metric that quantifies the model's ability to distinguish between presence and background locations (Valavi et al. 2021). Higher AUC values indicate better discriminatory power and overall model accuracy (Ab Lah et al. 2021; Wani et al. 2023). MaxEnt output maps for each scenario were classified into four potential habitat classes: not suitable (< 0.25), least suitable (0.25–0.50), moderately suitable (0.51–0.75), and highly suitable (> 0.75). Range change analysis was carried out following Wani, Abdul Rahim, et al. (2025). To assess the conservation implications of current and future habitat suitability, binary MaxEnt outputs (values < 0.5 classified as unsuitable and > 0.5 as suitable) for all scenarios were overlaid on a Protected Areas (PA) shapefile of the region. Using zonal statistics in ArcGIS 10.8, the area of suitable habitat (in km^2^) falling inside and outside the protected area network was calculated for each scenario.

## 3 Results and Discussion

### 3.1 Model Performance

**FIGURE 3:**
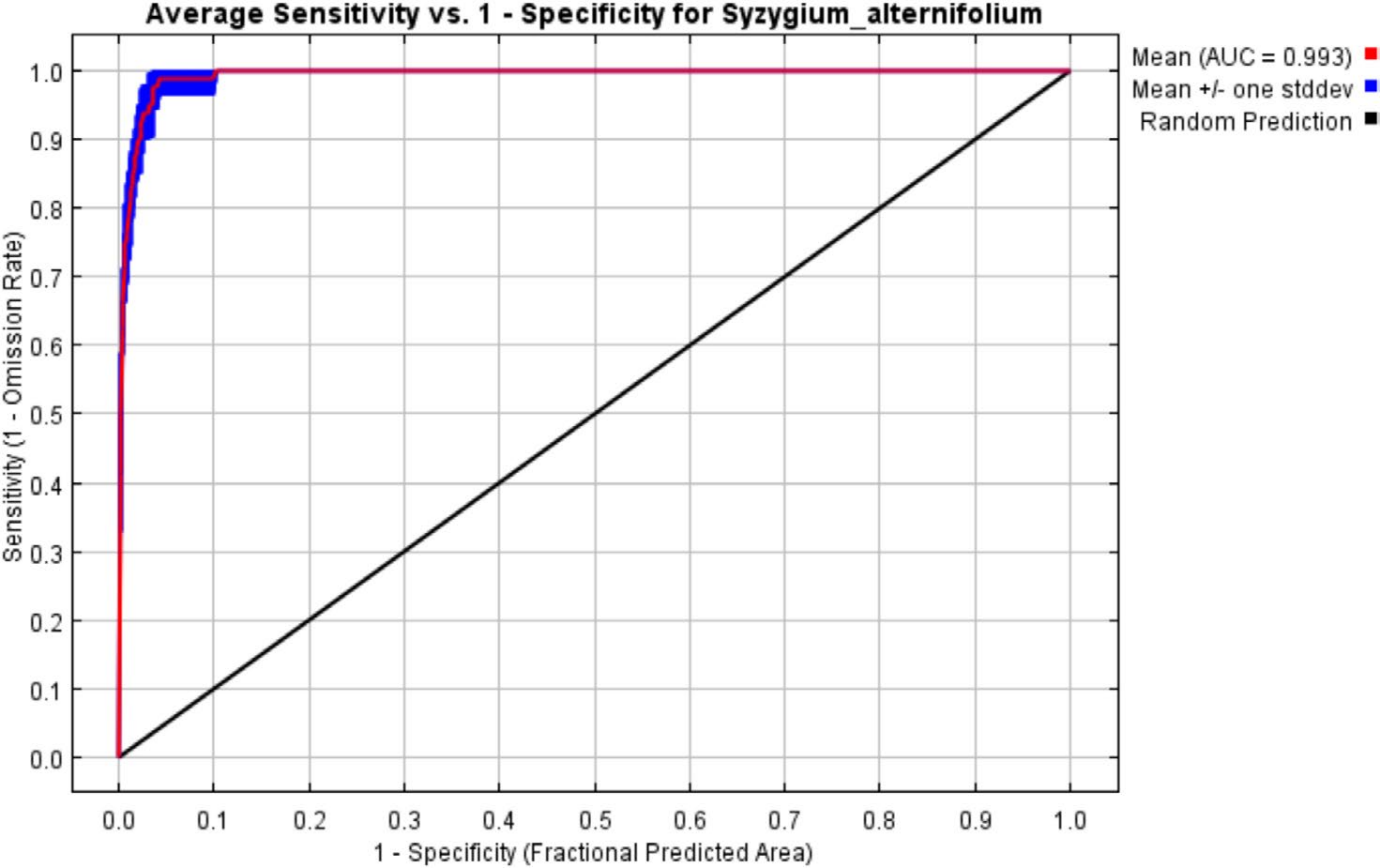
Receiver Operating Characteristic (ROC) curve for the MaxEnt model predicting the distribution of *Syzygium alternifolium*.

The MaxEnt model used for predicting the potential distribution of *S. alternifolium* demonstrated high predictive performance, as evidenced by the AUC value of 0.993. An AUC value close to 1 indicates an excellent ability of the model to differentiate between suitable and unsuitable habitats, thereby minimizing both omission (false negatives) and commission (false positives) errors (Khanghah et al. 2022; Elliott et al. 2024). The ROC (Receiver Operating Characteristic) curve illustrates the trade‐off between sensitivity (true positive rate) and 1‐specificity (false positive rate). In this case, the red line representing the model's average performance lies well above the diagonal black line, which indicates random prediction. This reflects the model's strong discriminatory power in separating presence from background points (Figure 3). The blue shaded region, representing standard deviation across replicates, shows that the model's performance is consistent across replicates. The sharp rise of the ROC curve near the origin further emphasizes the model's ability to correctly identify a high proportion of true presences with a very low false positive rate—indicating minimal omission errors.

### 3.2 Variable Importance

**FIGURE 4:**
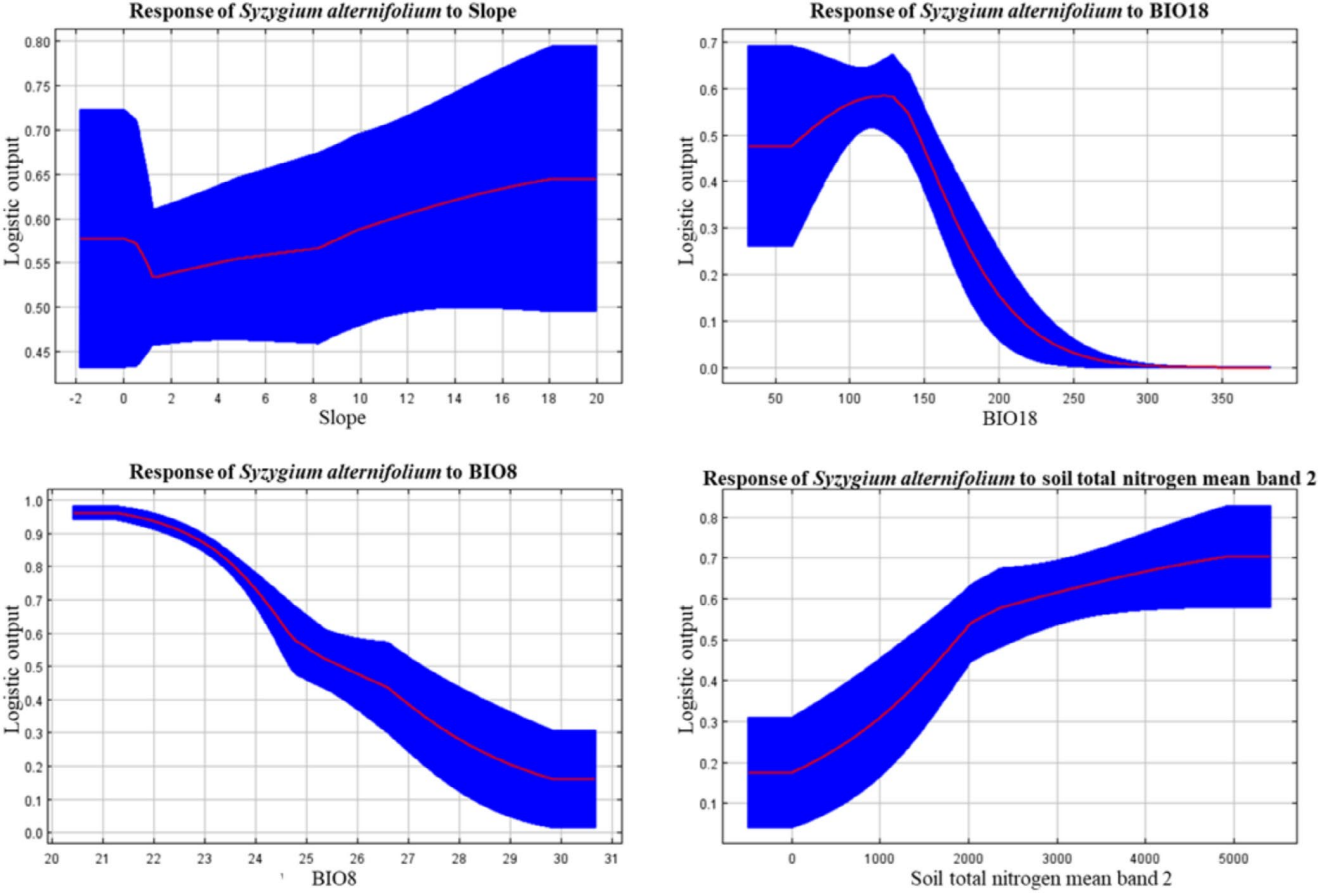
Response curves of key environmental predictors influencing the distribution of *Syzygium alternifolium* as modeled by the MaxEnt. The red lines represent the mean response, and the blue shaded areas indicate standard deviation.

The MaxEnt model identified several key environmental variables that contributed to the prediction of suitable habitats for *S. alternifolium* (Table 1). Among the 12 predictors used in the model, slope had the highest percent contribution (25.5%), followed by Bio18 (Precipitation of Warmest Quarter) (20.1%), Bio8 (Mean Temperature of Wettest Quarter) (14.0%), and soil total nitrogen (13.1%). These were the four most influential variables based on their percent contribution to the model. Soil organic carbon density and aspect also contributed moderately, with percent contributions of 6.9% and 6.6%, respectively. Climatic variables such as Bio2 (Mean Diurnal Range) and elevation had relatively lower percent contributions (6.0% and 3.9%, respectively), but their permutation importance was substantially higher, at 32.0% and 14.8%, respectively, indicating their strong influence on model accuracy when their values were randomized. Bio18 had the highest permutation importance (35.2%), followed by Bio2 (32.0%), further emphasizing their predictive power. Other variables like Bio11 (Mean Temperature of Coldest Quarter), soil clay content, and Bio10 (Mean Temperature of Warmest Quarter) showed very low contributions and permutation importance, suggesting limited influence on the model's output. Understanding the relationship between the geographical distribution of taxa and their environmental conditions is a key concept in ecology and conservation (Kaky et al. 2020). In the present study, the response curve analysis provides ecological insight into the environmental preferences of *S. alternifolium* and helps explain the species' limited and patchy distribution in the Eastern Ghats (Figure 4). Response curve of slope revealed that suitability slightly increases with slope. The curve remains relatively flat at lower slopes but gradually rises after a certain threshold, indicating preference for moderate to steep terrains. The gradual increase in suitability with slope reflects the species' association with undisturbed forested hill slopes, where soil drainage, reduced competition, and microclimate stability may support better regeneration. These areas are often less accessible and thus experience lower anthropogenic disturbance. Field observations and previous studies (Reddy and Ugle 2008) also report that *S. alternifolium* is found mostly on moderately steep to steep slopes within semi‐evergreen forest patches. These observations support the MaxEnt output and reinforce slope as a species‐specific habitat determinant. Response curve of Bio18 shows unimodal pattern suggesting that *S. alternifolium* requires a specific range of rainfall during the warm season for optimal growth. Excessive precipitation during this critical period might lead to soil leaching, pathogen proliferation, or water logging, all of which could reduce survival or seedling establishment.

**TABLE 1:** Percent contribution and permutation importance of environmental variables in determining the distribution of *Syzygium alternifolium*.

Variable	Percent contribution	Permutation importance
Slope	25.5	0.5
Bio18	20.1	35.2
Bio8	14	5.2
Soil_total_nitrogen‐mean_band_2 (5–15 cm)	13.1	1.7
Soil_organic‐carbon‐density_band_2 (5–15 cm)	6.9	2.3
Aspect	6.6	3.2
Bio2	6	32
Elevation	3.9	14.8
Bio11	1.9	2.2
Soil_claycontent_band_2 (5–15 cm)	1.8	1.9
Soil_claycontent_band_1 (0–5 cm)	0.2	0.4
Bio10	0	0.5

### 3.3 Current Distribution

**FIGURE 5:**
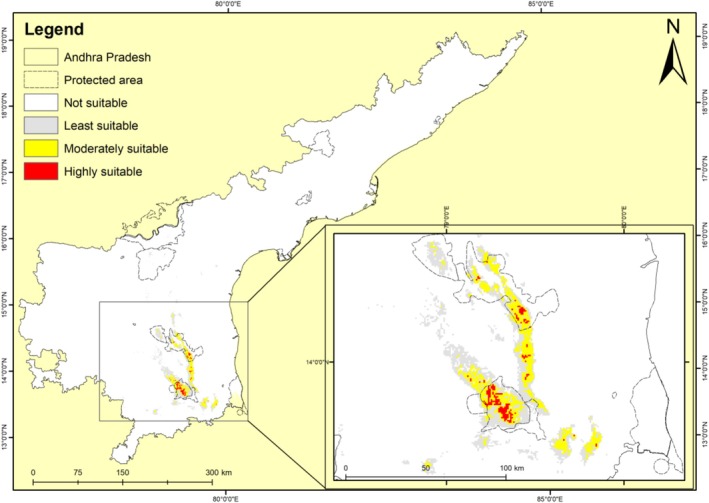
Current potential distribution of *Syzygium alternifolium* in Andhra Pradesh as predicted by MaxEnt.

The current habitat suitability map for *S. alternifolium* in Andhra Pradesh reveals a spatially restricted distribution, concentrated mainly in the southern Eastern Ghats (Figure 5). The highly suitable habitats (red areas) are confined to narrow patches along hill ranges, indicating the species' dependence on specific ecological and topographic conditions. These red zones are surrounded by moderately suitable habitats (yellow), which are comparatively more widespread, forming a contiguous belt along the hilly terrain. The least suitable areas (gray) and unsuitable zones (white) dominate the surrounding plains and fragmented landscapes, particularly in lowland regions. This predicted distribution corresponds well with the known endemic range of *S. alternifolium*, validating the model's accuracy and highlighting the species' limited ecological niche. The total suitable habitat for *S. alternifolium* under the current climate is estimated at 1262.39 km^2^, covering approximately 0.78% of the state's land area. Of this, 669.4 km^2^ comprising 53.02% of the total suitable area lies within the protected areas like Sri Venkateshwara National Park, Penusila Narasimhaswamy Wildlife Sanctuary, Sri Lankamalleswara Wildlife Sanctuary, Nagarjuna Sagar Srisailam Wildlife Sanctuary and Gundla Brahmeswaram Wildlife Sanctuary. A total of 592.99 km^2^ (46.97%) of the total suitable area falls outside the protected areas. Mohan and Lakshmi (2000) have reported that *S*. *alternifolium* is particularly found in Sri Venkateshwara Wildlife Sanctuary, inhabiting dry, slaty, rocky terrain and forms gregarious populations. Although the species is mainly distributed inside protected areas, it experiences high anthropogenic pressure in the form of unregulated harvesting for timber and medicines. Erratic flowering, bud infestation and flower predation by insects, poor fruit set, fruit predation by monkeys, bears, deer and wild boars, erratic rainfall following germination are some of the problems recorded in its natural habitat that have a profound effect on its regeneration capacity (Pattanaik et al. 2022). The hard seed coat and extremely short viability period (~7 days) of *S*. *alternifolium* seeds (Mohan and Lakshmi 2000) severely limit natural regeneration. In the absence of immediate favorable conditions, seeds lose viability rapidly, and the physical barrier posed by the seed coat further delays germination. Together, these traits make the species highly vulnerable to regeneration failure under changing disturbance and climatic conditions (Raju et al. 2014). Further, the effectiveness of protected areas is also a concern, as India has experienced significant habitat fragmentation with reports even from the protected areas (Wani, Singh, et al. 2025).

The predicted current distribution map illustrates that *S*. *alternifolium* occupies a narrow and limited ecological niche, predominantly within the southern Eastern Ghats of Andhra Pradesh. These high‐suitability areas align with regions of moderate to steep slopes, high seasonal rainfall (particularly during the warmest quarter), and nutrient‐rich soils, as also indicated by the variable contribution analysis. The clustering of highly suitable habitats along ridges and forested zones suggests that the species thrives in humid and forested hill environments. The presence of moderately suitable zones around the high‐suitability cores may represent buffer areas or zones for potential natural expansion, provided environmental pressures are mitigated. The limited distribution and patchiness in suitable habitats highlight the species' vulnerability to habitat degradation, fragmentation, and climate variability (Sękiewicz et al. 2024).

### 3.4 Future Distribution and Range Change

**FIGURE 6:**
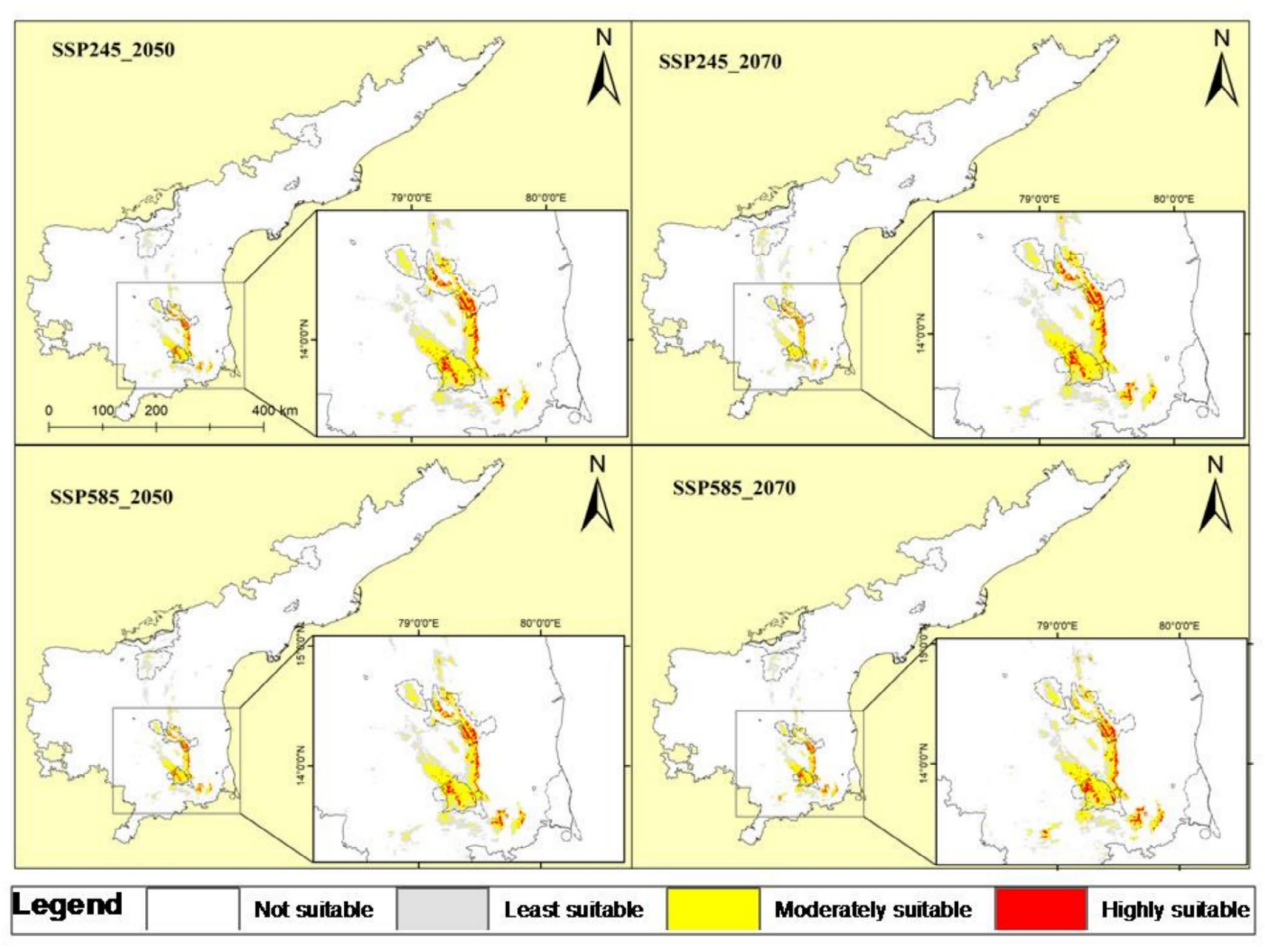
Projected future distribution of *Syzygium alternifolium* under different climate change scenarios for the years 2050 and 2070.

The future habitat suitability projections for *S. alternifolium* under four SSP scenarios, SSP245‐2050, SSP585‐2050, SSP245‐2070, and SSP585‐2070 indicate noticeable shifts in the spatial distribution and quality of suitable habitats (Figure 6). Future projections show a consistent expansion in suitable area under all climate scenarios: Under SSP245‐2050, the suitable area increases to 2556.86 km^2^ (102.54% increase) with 49.27% inside and 50.72% outside protected areas. By SSP245‐2070, suitable habitat further increases to 2717.22 km^2^ (115.24% gain), with a near‐equal distribution of area inside (49.74%) and outside (50.25%) protected areas. In the high‐emission SSP585‐2050 scenario, the total suitable habitat increases to 2638.27 km^2^ (108.99% increase), with 48.37% inside protected areas and 51.62% outside (Table 2). The largest expansion is seen under SSP585‐2070, reaching 2813.45 km^2^, a 122.87% increase, but only 43.99% of this falls within protected areas, with 56.0% falling outside. These findings indicate both a net expansion of suitable habitats under all climate projections and a progressive shift in habitat distribution outside current protected area boundaries, especially under the more severe SSP585 scenarios. This suggests that much of the future suitable habitat is expected to occur outside current protected area networks, potentially in human‐dominated or unregulated landscapes.

**TABLE 2:** Projected changes in suitable habitat area for *Syzygium alternifolium* under current and future climate scenarios (SSP245 and SSP585 for 2050 and 2070).

Scenario	Total suitable area (km^2^)	%age	Range change (%)	Suitable area inside protected area	%age	Suitable area outside protected area	%age
Current	1262.39	0.78		669.4	53.02	592.99	46.97
SSP245‐2050	2556.86	1.58	↑102.54	1259.9	49.27	1296.96	50.72
SSP245‐2070	2717.22	1.68	↑115.24	1312.5	49.74	1325.77	50.25
SSP585‐2050	2638.27	1.63	↑108.99	1276.3	48.37	1361.97	51.62
SSP585‐2070	2813.45	1.74	↑122.87	1237.9	43.99	1575.55	56.0

**FIGURE 7:**
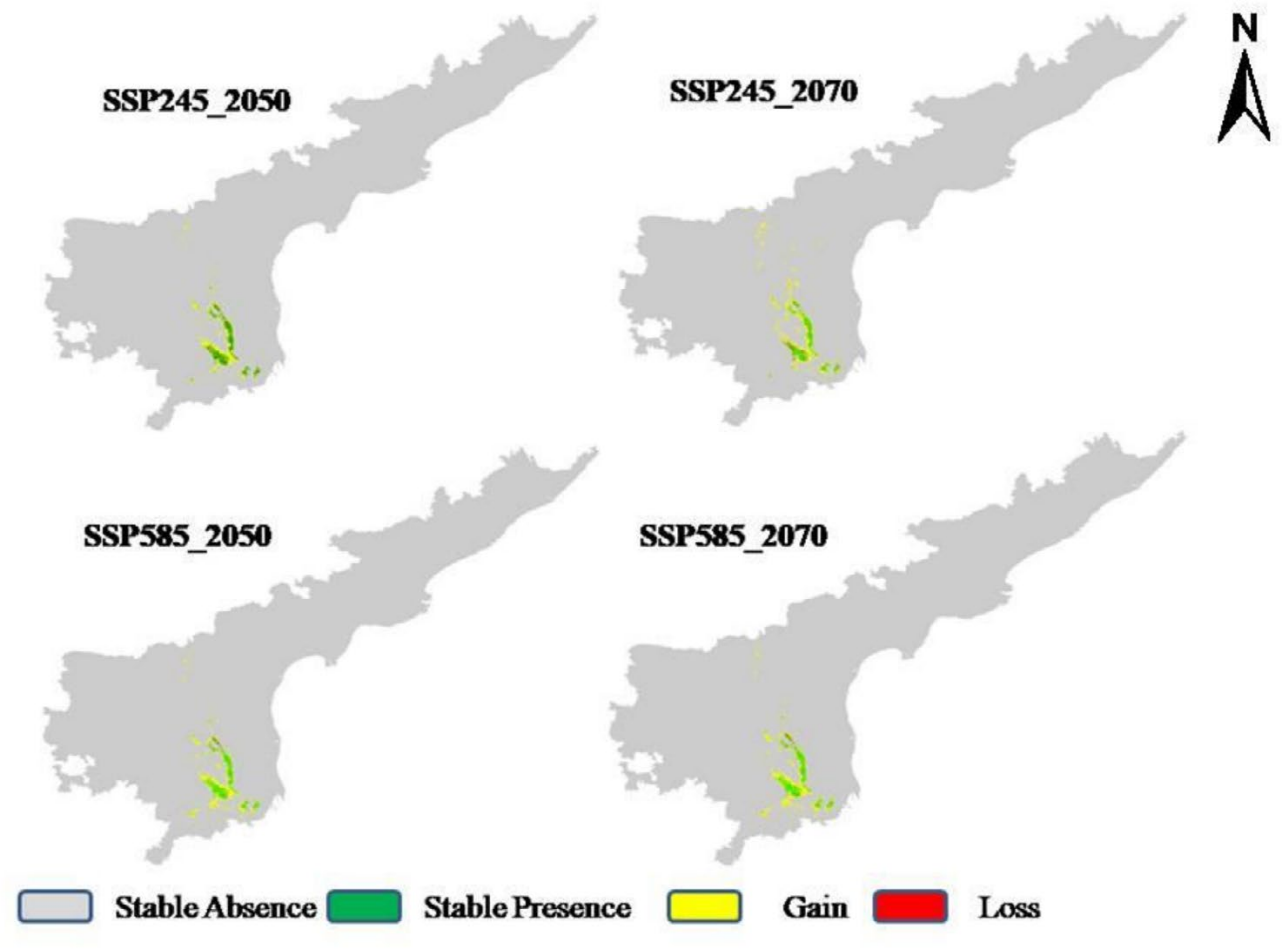
Predicted range dynamics of *Syzygium alternifolium* under future climate scenarios (SSP245 and SSP585 for 2050 and 2070).

The range change maps for *S. alternifolium* reveal a consistent trend of habitat expansion across all future projections (Figure 7). Areas of stable presence (green) remain largely concentrated in the southern Eastern Ghats, suggesting climatic resilience of current core habitats. Notably, substantial habitat gain (yellow) is projected, particularly under the high‐emission scenario SSP585‐2070 (1626.5 km^2^), indicating a potential northward and eastward shift in the species' suitable range (Figure 7). The mid‐century projections under both SSP245 and SSP585 show moderate expansion (1419 and 1368 km^2^, respectively), with new suitable areas emerging adjacent to the current range, while the late‐century scenarios predict more pronounced gains into previously unsuitable regions. Similar spatial shifts have been observed in other taxa responding to global warming (Lenoir and Svenning 2015; Pélissié et al. 2022; Yang et al. 2024). High‐emission scenarios like SSP585 have been shown to increase habitat suitability of some plant species in previously cooler or drier regions (Chowdhary et al. 2025). The emergence of new suitable areas in previously marginal zones suggests potential migration corridors or reintroduction sites; however, these areas may lack the specific microhabitat conditions or soil fertility required for successful establishment unless. Whether these newly suitable habitats will be colonized depends on the species' dispersal capacity and ecological interactions, coupled by active restoration or assisted migration efforts (Rivera et al. 2021; da Silva et al. 2024). Habitat loss (red) is minimal across all scenarios, implying that most of the currently suitable areas are likely to persist under future climates. Overall, across all future scenarios, some previously unsuitable or least suitable areas transition into moderately or highly suitable zones, suggesting climatic changes may render new areas suitable, while others may become less hospitable.

Climatic shifts may offer new opportunities for natural expansion and assisted colonization of *S*. *alternifolium*. But the protection gap could expose future populations to threats such as deforestation, agricultural encroachment, and infrastructure development. To ensure long‐term species persistence, it is essential to identify and prioritize future suitable zones outside protected areas for conservation interventions, promote landscape‐level planning and community‐managed reserves in high‐suitability zones beyond current PAs, enhance connectivity between existing and future habitats to facilitate natural dispersal, and monitor climate‐vulnerable populations within current PAs that may become less suitable under future conditions. Thus, climatic changes may temporarily benefit *S. alternifolium* by expanding its potential range; spatial mismatch between future suitable habitats and protected areas poses a critical conservation challenge that must be proactively addressed.

## 4 Conclusion

This study successfully employed the MaxEnt model to predict the current and future distribution of the endemic tree species *S. alternifolium* in Andhra Pradesh under various climate change scenarios. The model demonstrated excellent predictive performance, indicating high reliability in estimating habitat suitability. Results revealed that the species' distribution is strongly influenced by environmental variables, with slope, precipitation of the warmest quarter (Bio18), temperature of the wettest quarter (Bio8), and soil total nitrogen emerging as the most significant predictors. Response curves confirmed the species' preference for moderately steep, nitrogen‐rich, and well‐drained habitats, with cool, moist conditions during the monsoon and moderate rainfall during summer. The current distribution is highly restricted and fragmented within the Eastern Ghats. However, future projections suggest a significant expansion in suitable habitats, particularly under high‐emission scenarios. Notably, much of this expansion is expected to occur outside existing protected areas, raising concerns over increased exposure to anthropogenic threats.

To safeguard the species, it is essential to prioritize conservation of currently suitable habitats, particularly those identified as highly suitable, through effective management of protected areas and forest reserves. The designation of Ecologically Sensitive Zones (ESZs) around core populations could further buffer against external disturbances. Simultaneously, areas predicted to become suitable in the future, especially those beyond existing protection, should be identified and monitored, and potentially incorporated into conservation strategies such as community forests or biosphere reserves. Reforestation and habitat enrichment in moderately suitable areas can support natural range expansion, and in emerging zones under future climates, assisted colonization may be necessary. Given the species' sensitivity to slope, soil nitrogen, and specific climate variables, efforts to reduce landscape degradation such as erosion, overgrazing, and deforestation are crucial. Long‐term monitoring using permanent plots, along with eco‐physiological and genetic studies, should be promoted to understand the adaptive capacity and resilience mechanisms of *S. alternifolium*. Finally, it is vital to integrate MaxEnt modeling outputs into biodiversity action plans, forest working plans, and climate adaptation strategies at both state and regional levels.

## Author Contributions

**Abdul Rahim PP:** conceptualization (equal), data curation (lead), formal analysis (equal), investigation (lead), methodology (lead), software (lead), writing – original draft (lead), writing – review and editing (equal). **Zishan Ahmad Wani:** formal analysis (equal), methodology (supporting), writing – original draft (supporting), writing – review and editing (supporting). **Javid Ahmad Dar:** conceptualization (equal), formal analysis (equal), methodology (equal), software (equal), supervision (lead), validation (lead), writing – original draft (equal), writing – review and editing (equal). **Subashree Kothandaraman:** formal analysis (supporting), investigation (supporting), methodology (supporting), writing – review and editing (supporting). **Yashwant S. Rawat:** validation (supporting), writing – review and editing (supporting).

## Funding

The authors have nothing to report.

## Conflicts of Interest

The authors declare no conflicts of interest.

## Supporting information


**Table S1:** Occurrence points of *Solanum viarum* used for the modeling.

**Table S2:** Correlation among the variables.

## Data Availability

All data supporting the findings of this study are included within the manuscript and its Supporting Information.
